# Effects of a psychological capital intervention on emergency nurses’ work engagement and turnover intention: A randomized controlled trial

**DOI:** 10.1097/MD.0000000000048971

**Published:** 2026-05-22

**Authors:** Kun Sun, Xiaoyan Gong, Guoping Yuan

**Affiliations:** a Nursing Department, Sir Run Run Shaw Hospital, Zhejiang University School of Medicine, Hangzhou, China.

**Keywords:** emergency department, nurse, psychological capital intervention, turnover intention, work engagement

## Abstract

**Background:**

Emergency nurses are exposed to high occupational stress, which leads to decreased psychological capital, reduced work engagement, and high turnover intention. Evidence on targeted psychological capital intervention (PCI) for this population remains limited.

**Methods:**

A parallel randomized controlled trial was conducted. A total of 122 emergency nurses from a tertiary hospital in Zhejiang Province were randomly assigned to the intervention group (6-week PCI, n = 61) or the control group (routine training, n = 61). Outcomes were measured at baseline (T0), post-intervention (T1), 1 month (T2), and 3 months (T3) using the Psychological Capital Questionnaire, Utrecht Work Engagement Scale, and Turnover Intention Scale.

**Results:**

Baseline characteristics and outcome scores were comparable between groups (all *P* > .05). At T1 to T3, the intervention group had significantly higher Psychological Capital Questionnaire and Utrecht Work Engagement Scale scores and lower Turnover Intention Scale scores than the control group (all *P* < .001). Psychological capital was positively correlated with work engagement (*R* = 0.604, *P* < .001) and negatively correlated with turnover intention (*r* = ‐0.624, *P* < .001).

**Conclusion:**

This 6-week PCI program significantly improves psychological capital and work engagement, and reduces turnover intention among emergency nurses, with effects sustained for at least 3 months. These findings support the utility of PCI as a psychological management strategy for emergency nursing staff.

## 1. Introduction

The emergency department (ED), as the frontline for treating critically ill patients, is characterized by a high-risk environment, frequent complaints, and numerous safety hazards. Nurses in this setting contend with complex and rapidly changing patient conditions, time-sensitive interventions, and heightened emotional demands from anxious or distressed family members, which often lead to unrealistic expectations for care delivery. These factors contribute to chronic physical and mental strain among emergency nurses, manifesting as significant psychological fatigue, reduced work engagement, and diminished self-efficacy.^[1]^ A number of studies have shown that between 27% and 46.9% of ED nursing staff report experiencing high levels of occupational stress,^[2,3]^ which not only compromises their well-being but also threatens the quality of care and patient safety. With the increasing emphasis on patient-centered care, nurses’ roles are evolving beyond technical tasks to encompass more creative and integrative service provision, necessitating higher levels of professional competence. Work engagement and turnover intention, as key indicators of professional competence, directly affect a hospital’s competitive advantage.^[4,5]^ As such, the development of effective interventions to enhance emergency nurses’ psychological capital, work engagement, and retention has become an urgent priority.

Psychological capital (PsyCap), a key concept in positive organizational behavior introduced by Luthans et al in 2007, encompasses 4 dimensions: self-efficacy, hope, optimism, and resilience.^[6]^ The psychological capital intervention (PCI) model is designed to enhance individuals’ work performance by cultivating these positive psychological resources. Recent studies across various fields, including management, education, and clinical medicine, have demonstrated the efficacy of PsyCap in reducing burnout and improving job satisfaction.^[7–9]^ For example, PsyCap has been shown to alleviate work-related exhaustion among nurses.^[10]^ Additionally, Brunetto et al found that nurses with higher levels of PsyCap tend to exhibit lower turnover intention and stronger organizational commitment. While research on PsyCap has evolved from focusing on individual-level outcomes to examining its impact within team and organizational contexts.^[11–14]^ Moreover, existing studies have predominantly focused on general nursing populations, leaving the unique challenges faced by emergency nurses underexplored. In light of this, designing a PCI-based group intervention specifically tailored to the ED setting could address this research gap and offer evidence-based strategies to improve care quality and stabilize the workforce.

## 2. Methods

This study was approved by the Research Ethics Committee of Sir Run Run Shaw Hospital, Zhejiang University School of Medicine (approval No. 20250057). The study was performed in accordance with the ethical standards laid down in the 1964 Declaration of Helsinki and its later amendments. Written informed consent was obtained from all participants prior to enrollment. All participants were informed of the study purpose, procedures, potential risks, benefits, and their right to withdraw at any time without penalty.

This study investigates the effects of a PCI-based group intervention on emergency nurses’ psychological capital, work engagement, and turnover intention. The findings are intended to inform clinical nursing human resource management practices and contribute to the localized application of PsyCap theory within healthcare settings.

### 2.1. Sample size calculation

The sample size was calculated using PASS 15.0 software (NCSS Statistical Software [NCSS, LLC], Kaysville) based on the primary outcome (total Psychological Capital Questionnaire [PCQ] score). The effect size (*d* = 1.23) was obtained from our pilot study conducted in the same ED of this tertiary hospital before formal recruitment, which reflected the actual intervention effect in this target population. The parameters were set as follows: α = 0.05 (two-tailed), power = 80%, and a 20% attrition rate for longitudinal follow-up. The minimal required sample size was 120 participants. To ensure sufficient statistical power for repeated-measures analysis and compensate for potential dropout, the final sample size was expanded to 122 participants, with 61 nurses in each group. We acknowledge that this effect size is relatively large compared with general psychosocial interventions, which may limit the generalizability of the power calculation to other populations; this limitation is further discussed in Section 5.

### 2.2. Study participants and sampling

A total of 122 participants were randomized and included in the intention-to-treat (ITT) analysis. Attrition and missing data were monitored at all 4 time points: T0 (baseline), T1 (post-intervention), T2 (1 month), and T3 (3 months). No participants were lost to follow-up; the missing data rate was <5% at each time point across all outcomes. Missing values were handled using multiple imputation with 10 imputed datasets. The imputation model included age, work experience, professional title, baseline scores of psychological capital, work engagement, turnover intention, and intervention attendance as auxiliary variables to improve imputation precision. All randomized participants were retained in the ITT analysis regardless of compliance (demographics in Table 1).

**Table 1:** General information of nurses in the emergency department.

Item	Group	Category	Number	Percentage (%)	χ^2^	*P*
Sex	Intervention (n = 61)	Male	12	19.7	0.082	.775
		Female	49	80.3		
	Control (n = 61)	Male	11	18.0		
		Female	50	82.0		
Age	Intervention (n = 61)	22–25 yr	1	1.6	0.156	.986
		26–35 yr	46	75.4		
		36–45 yr	13	21.3		
		>45 yr	1	1.6		
	Control (n = 61)	22–25 yr	1	1.6		
		26–35 yr	47	77.0		
		36–45 yr	12	19.7		
		>45 yr	1	1.6		
Edu	Intervention (n = 61)	Associate degree	3	4.9	0.281	.868
		Bachelor’s degree	55	90.2		
		Master’s/doctorate degree	3	4.9		
	Control (n = 61)	Associate degree	2	3.3		
		Bachelor’s degree	56	91.8		
		Master’s/doctorate degree	3	4.9		
Marital	Intervention (n = 61)	Single	22	36.1	0.143	.931
		Married	38	62.3		
		Divorced	1	1.6		
	Control (n = 61)	Single	23	37.7		
		Married	37	60.7		
		Divorced	1	1.6		
Work_age	Intervention (n = 61)	<5 yr	5	8.2	0.419	.979
		6–10 yr	32	52.5		
		11–15 yr	21	34.4		
		16–20 yr	1	1.6		
		>20 yr	2	3.3		
	Control (n = 61)	<5 yr	4	6.6		
		6–10 yr	33	54.1		
		11–15 yr	22	36.1		
		16–20 yr	1	1.6		
		>20 yr	1	1.6		
Professional title	Intervention (n = 61)	Primary	38	62.3	0.085	.771
		Intermediate	23	37.7		
		Senior	0	0		
	Control (n = 61)	Primary	39	63.9		
		Intermediate	22	36.1		
		Senior	0	0		

*Note*: There were no significant differences in all baseline characteristics between the 2 groups (all *P* > .05), indicating good comparability.

Inclusion criteria: valid nursing license and ≥1 year of ED experience at the study hospital; no history of psychological intervention (e.g., counseling, psychotherapy) within the past 3 months; voluntary participation and ability to complete all follow-up assessments; no plans to resign, take extended leave (≥2 weeks), or attend external training during the study period.

Exclusion criteria: diagnosis of mental illness (e.g., depression, anxiety disorder) or severe physical diseases (e.g., malignancies, severe cardiovascular conditions) affecting participation; cognitive impairments that would prevent independent completion of questionnaires; voluntary withdrawal or loss of contact during the study. The study ultimately included 128 cases, with 6 cases excluded, and data from 122 cases were used for subsequent statistical analysis.

Randomization and allocation concealment: eligible participants were stratified based on 3 variables: “age (26–35 years/36–45 years/other),” “work experience (6–10 years/11–15 years/other),” and “professional title (primary/intermediate/senior).” Within each stratum, participants were randomly assigned to the intervention or control group at a 1:1 ratio using a computer-generated random number table (Excel’s RAND function). Random sequences were sealed in opaque, sequentially numbered envelopes by an independent statistician not involved in participant recruitment. Envelopes were opened only after confirming eligibility and obtaining informed consent to ensure allocation concealment and prevent selection bias.

Ethics approval: No. 20250057 (Research Ethics Committee of Sir Run Run Shaw Hospital, Zhejiang University School of Medicine); written informed consent was obtained from all participants prior to enrollment. This randomized controlled trial was initially implemented as a hospital-based quality improvement project and was not prospectively registered in a public clinical trial registry. The study was conducted in strict accordance with the CONSORT statement for randomized controlled trials.

#### 2.2.1. Research team formation

The research team consisted of 7 members, each responsible for specific tasks, with all members affiliated with or collaborating with the study hospital:

The head nurse of the ED oversaw team formation, organized protocol revision meetings, and supervised intervention implementation and quality control.

A chief physician from the ED participated in protocol revisions and provided clinical guidance on integrating intervention content into emergency practice.

An associate chief physician from the psychiatry department conducted content review of the intervention and provided standardized psychological counseling training (2 sessions, 4 hours total) for the research team.

Two senior nurses from the ED, each with ≥5 years of experience and a national Level-2 Psychological Counselor certificate, delivered face-to-face intervention, addressed participant questions, and recorded implementation details (e.g., attendance, feedback).

The principal investigator, affiliated with the nursing department, coordinated protocol development, intervention scheduling, questionnaire distribution/collection, and data collation.

An independent statistician, collaborating with the hospital’s medical statistics department, managed randomization, allocation concealment, and statistical analysis to minimize researcher bias.

#### 2.2.2. Preliminary preparation

One week prior to the intervention (T0), baseline assessments were conducted among all 122 participants. These assessments included: a self-designed demographic questionnaire; the PCQ; the Work Engagement Scale (UWES); and the Turnover Intention Scale (TIS). All questionnaires were completed independently by participants in a quiet conference room within the ED, with research team members available to answer any questions regarding the completion process (without providing guidance on specific answers).

#### 2.2.3. Development of the psychological capital intervention training program

The intervention protocol was developed based on Luthans et al’s PCI model. Following a systematic review of relevant literature and 3 rounds of collaborative discussions among the research team (including 2 senior critical care nursing specialists from the hospital), a modular intervention program was created, focusing on 4 core themes: “cultivating hope,” “fostering optimism,” “enhancing self-efficacy,” and “building resilience.”

The preliminary protocol underwent pilot testing with 10 emergency nurses from the same department to assess feasibility and acceptability. Revisions based on pilot feedback included: reducing the duration of the “Pressure Mapping” exercise from 30 minutes to 20 minutes to prevent disruption to nurses’ work schedules; simplifying the “Strength Bombardment” steps (e.g., providing a list of common nursing strengths to facilitate expression); and incorporating emergency-specific cases (e.g., managing stress after a failed rescue) to enhance relevance. The final protocol was approved by the expert group, and a Standardized Intervention Manual was created (including session scripts, activity templates, and contingency plans) to ensure consistency in intervention delivery.

#### 2.2.4. Implementation of the psychological capital intervention training program

##### 2.2.4.1. Intervention group

The 6-week intervention was conducted from February to April 2024, with weekly sessions held every Thursday from 15:00 to 16:30 in the ED demonstration classroom (avoiding peak work hours). Prior to implementation, all research team members (interventionists and quality control personnel) underwent standardized training in psychological capital theory, session protocols, data collection, and quality control measures, and passed a competency assessment (score ≥ 85/100) to ensure fidelity.

A quality control supervisor (the head nurse of the ED) monitored the intervention process: recording attendance (with reasons for absences); video-recording 30% of sessions (randomly selected) for later compliance review; offering individualized follow-up support for participants who missed ≤2 sessions (e.g., sending session materials via WeChat and arranging 30-minute one-on-one supplementary sessions). Participants who missed >2 sessions were considered “non-compliant” but were still included in the ITT analysis.

The intervention consisted of 4 core modules (detailed in Table 2).

**Table 2:** Implementation plan for psychological capital intervention training.

Training sequence (time)	Theme	Objectives	Content	Activity methods	Operation standards
1st session (1st week)	Training on theoretical knowledge of psychological capital meeting by fate	① Create a harmonious team atmosphere; ② establish group rules and sign a group contract; ③ understand the definition, dimensions, and significance of psychological capital for emergency nurses.	① Introduce study objectives, intervention process, and group rules; ② “Musical Chairs” icebreaker; ③ lecture on psychological capital theory (with cases of emergency nurses’ psychological challenges).	Warm-up game: musical chairs (15 min) ; lecture (30 min); group contract signing (15 min)	① Musical chairs: stop music at fixed intervals, nurses without chairs share 1 work expectation; ② lecture uses PPT with local cases (e.g., how optimism helps handle patient complaints); ③ contract includes attendance, confidentiality, and participation commitments, signed by all.
2nd session (2nd week)	Embrace hope march towards the sun	① Help nurses discover personal and peers’ work strengths; ② enhance work mission sense; ③ set SMART work goals.	① “Embrace and Praise” activity; ② discussion on “most meaningful work experience in 1 year”; ③ guide setting 3-month goals (e.g., mastering 1 emergency skill).	“Embrace and Praise” game (20 min); group discussion (25 min); goal-setting worksheet (10 min)	① Praise must be specific (e.g., “You calmly handled hypoglycemia last week”); ② goals are recorded in worksheets with monthly self-assessment; ③ incentives: notebooks with hospital emergency logo.
3rd session (3rd week)	Cultivate an optimistic spirit	① Master Ellis ABC emotional regulation model; ② correct irrational work beliefs; ③ cultivate positive coping attitudes.	① Share work stress events; ② analyze events with ABC model; ③ role-play to practice changing beliefs.	Case sharing (20 min); ABC model teaching (20 min); role-play (15 min)	① 3 to 4 nurses share cases, others analyze; ② ABC model is visualized on a whiteboard; ③ role-play includes “nurse,” “patient family” roles, observers give feedback.
4th session (4th week)	Improve self-efficacy	① Recall successful work experiences; ② recognize professional strengths; ③ learn to seek social support.	① “Proud Moment” sharing; ② “Strength Bombardment” activity; ③ discuss support-seeking methods.	Focus group discussion (25 min); “Strength Bombardment” (20 min); support mapping (10 min)	① “Proud Moment” includes specific details (time, process); ② strengths are work-related (e.g., “good at venous puncture”); ③ support mapping records colleagues’ contact info.
5th session (5th week)	Enhance resilience	① Identify work stressors; ② master 2 to 3 stress relief techniques; ③ improve emergency coping ability.	① Draw “Pressure Map”; ② teach relaxation training (4-7-8 breathing); ③ “Breakthrough Challenge” simulation.	“Pressure Map” drawing (20 min); relaxation training (15 min); scenario simulation (20 min)	① “Pressure Map” categorizes stressors (workload, doctor–patient relationship); ② relaxation training uses guided audio (5 min); ③ simulation involves emergency scenarios (e.g., cardiac arrest).
6th session (6th week)	Share gains and self-management	① Summarize intervention gains; ② establish peer support system; ③ develop post-intervention plans.	① Share psychological and behavioral changes; ② establish a WeChat support group; ③ formulate self-management plans.	Experience sharing (30 min); WeChat group establishment (5 min); plan formulation (20 min)	① Sharing includes specific changes (e.g., “I use breathing to cope with night shift anxiety”); ② group is moderated by interventionists (answers questions 3 times/week); ③ plans include weekly self-assessments.

##### 2.2.4.2. Control group

The control group received only routine emergency training, consistent with the hospital’s regular schedule: emergency skills training (1 session every 6 months, 2 hours per session, e.g., trauma first aid, ventilator operation); infection control training (1 session per quarter, 2 hours per session). The total training hours (9 hours over 6 weeks) matched those of the intervention group to minimize confounding effects from “training time.” Nurses in the control group were not allowed to participate in the intervention group’s activities. If they accidentally encountered intervention content, they remained in the control group for analysis (recorded as a protocol deviation). After the 3-month follow-up (T3), the control group was offered a 2-hour condensed version of the PCI intervention (focused on emotional regulation and stress relief) to ensure ethical fairness.

#### 2.2.5. Quality control

Intervention fidelity was strictly monitored throughout all sessions. A standardized fidelity checklist developed according to the original PCI model and the study-specific intervention manual was used for assessment. The checklist included core components such as session content delivery, activity implementation, time control, facilitator adherence, and participant engagement.

In total, 30% of intervention sessions were randomly selected and video-recorded for fidelity review. Two independent trained reviewers rated all recorded sessions using the fidelity checklist. Inter-rater reliability was excellent (intraclass correlation coefficient = 0.92). The predefined fidelity threshold was ≥90% adherence to the manual; all reviewed sessions met or exceeded this standard.

Any minor protocol deviations were documented immediately and corrected in subsequent sessions. Additional standardized training was provided if needed to ensure consistent and high-quality delivery of the PCI program.

### 2.3. Primary and secondary outcomes

*Primary outcome*: total score of the PCQ. *Secondary outcomes*: total score of the UWES and total score of the TIS.

#### 2.3.1. Demographic questionnaire for emergency department nurses

A self-designed demographic questionnaire was developed to gather essential background information, including gender, age, educational attainment, years of nursing experience, professional title, and marital status. The questionnaire was revised based on expert consultation (content validity index = 0.92) and pilot testing (completion time < 5 minutes) to ensure both comprehensiveness and brevity.

#### 2.3.2. Twenty-four-item Psychological Capital Questionnaire

The study utilized the 24‑item PCQ developed by Luthans,^[6]^ which assesses 4 dimensions of psychological capital: self-efficacy, hope, resilience, and optimism, with 6 items allocated to each dimension. In Luthans’ original validation study, the scale demonstrated excellent reliability (Cronbach α = 0.80) and maintained strong internal consistency in the current Chinese context (α = 0.945). The reliability coefficients for each dimension were as follows: self-efficacy (α = 0.870), hope (α = 0.894), resilience (α = 0.877), and optimism (α = 0.870). The questionnaire includes a total of 24 items, evenly distributed across the 4 dimensions (6 items per dimension). Responses were measured on a 6-point Likert scale, where 1 = Strongly Disagree, 2 = Disagree, 3 = Slightly Disagree, 4 = Slightly Agree, 5 = Agree, and 6 = Strongly Agree. Higher total scores reflect higher levels of psychological capital.

#### 2.3.3. Utrecht Work Engagement Scale

The Work Engagement Scale employed in this study was adapted from the UWES, developed by Schaufeli et al.^[15]^ The Chinese version of the scale, translated and revised by Zhang et al,^[16]^ was utilized to assess the work engagement of nurses. The UWES is one of the most widely used instruments for measuring work engagement. The 17-item questionnaire comprises 3 dimensions: vigor (items 1, 4, 7, 10, 13, 16), dedication (items 2, 5, 8, 11, 14), and absorption (items 3, 6, 9, 12, 15, 17). Responses were recorded on a 5-point Likert scale, with the following options: 1 = Never, 2 = Rarely, 3 = Occasionally, 4 = Frequently, 5 = Always. Higher composite scores indicate higher levels of work engagement. The scale demonstrated excellent reliability, with Cronbach alpha coefficients of 0.865 for vigor, 0.878 for dedication, and 0.868 for absorption. The overall reliability of the scale was 0.937, reflecting strong internal consistency and high psychometric validity.

#### 2.3.4. Turnover Intention Scale

The TIS was originally developed by Michaels and Spector in 1982.^[17]^ The scale consists of 6 items, organized into 3 dimensions: Turnover Intention I, Turnover Intention II, and Turnover Intention III. Responses were measured on a 4-point Likert scale, with the following options: “Frequently” = 4 points, “Occasionally” = 3 points, “Rarely” = 2 points, and “Never” = 1 point. Higher total scores indicate a stronger intention to leave. The Chinese version of the TIS demonstrated acceptable psychometric properties, with a Cronbach α coefficient of 0.773, indicating adequate internal consistency, and a content validity index of 0.68.

### 2.4. Data collection methods

Data were collected at 4 time points to assess the sustainability of the intervention effects:

T0 (1 week before intervention): demographic questionnaire, PCQ, UWES, and TIS.

T1 (immediately after intervention, 6th week): PCQ, UWES, and TIS.

T2 (1 month after intervention): PCQ, UWES, and TIS.

T3 (3 months after intervention): PCQ, UWES, and TIS.

Electronic questionnaires were distributed via Wen Juan Xing, a validated Chinese online survey platform, with a unique QR code assigned to each participant to prevent duplicate submissions.

### 2.5. Statistical analysis

Data were analyzed using SPSS 25.0 (IBM Corp., Armonk). Categorical variables were presented as frequencies and percentages. Continuous variables were tested for normality using the Shapiro–Wilk test; normally distributed data were reported as mean ± standard deviation with 95% confidence intervals (95% CI), and non-normally distributed data were reported as median and interquartile range. Between-group differences in baseline demographic characteristics were compared using the Chi-square test (χ^2^) for categorical variables and independent samples *t* test for normally distributed continuous variables.

For the primary and secondary outcomes across 4 time points (T0, T1, T2, and T3), a two-way repeated-measures analysis of variance was conducted to evaluate the main effects of group (intervention vs control), time, and group × time interaction effects. Mauchly test was used to examine the assumption of sphericity; if violated, the Greenhouse–Geisser correction was applied to adjust degrees of freedom and *P*-values. Post hoc pairwise comparisons were performed with the Bonferroni correction to control for Type I error. Partial eta squared (partial *η*^2^) was reported as a measure of effect size for interaction effects.

**Figure 1.**
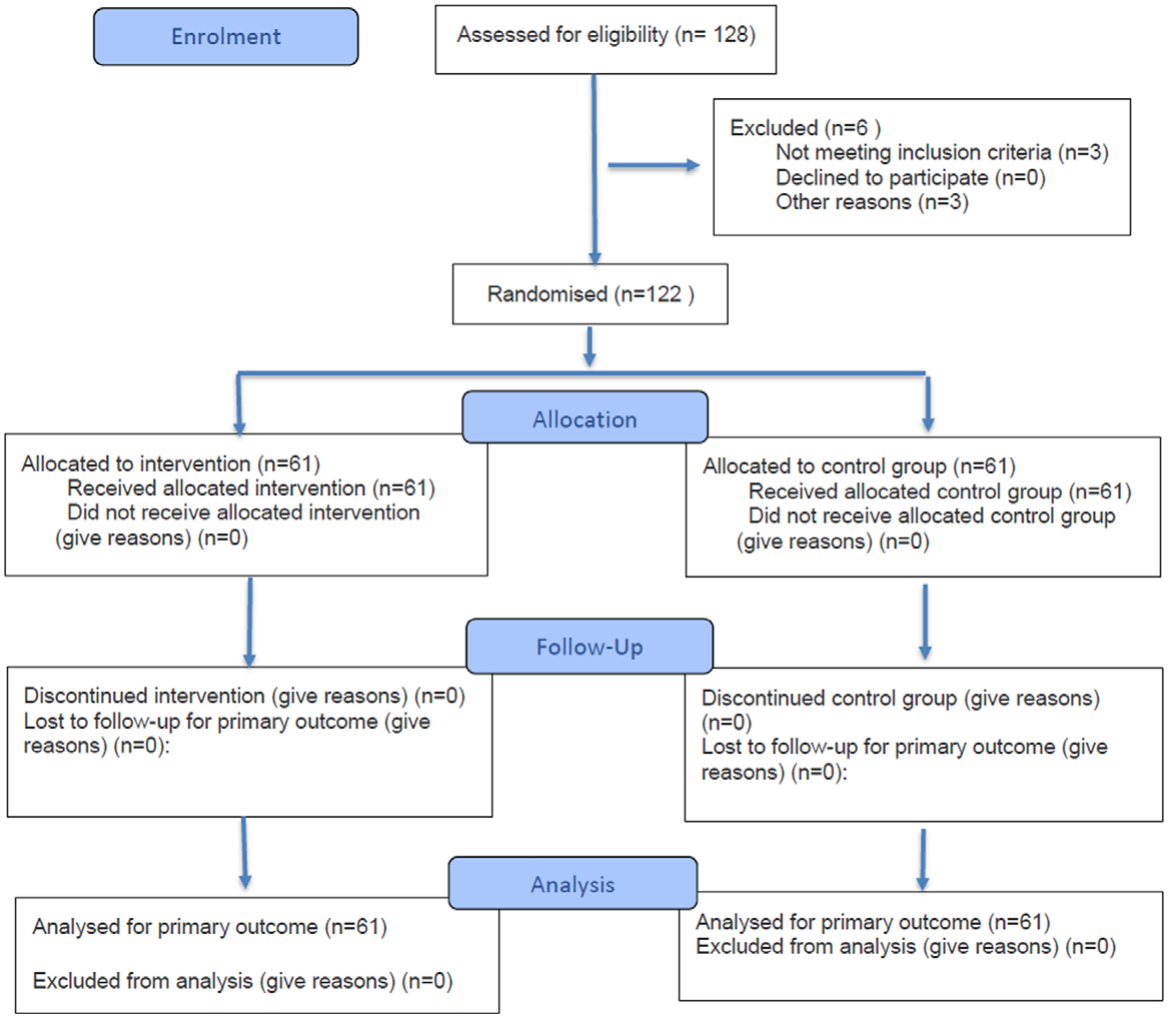
CONSORT 2025 flow diagram of participant recruitment, randomization, allocation, follow-up, and analysis. A total of 128 emergency nurses were assessed for eligibility; 6 were excluded. One hundred twenty-two participants were randomly assigned (1:1) to the intervention group (n = 61) or control group (n = 61). All randomized participants completed the intervention and follow-up; no participants were lost to follow-up or excluded after randomization. All 122 participants were included in the intention-to-treat (ITT) analysis.

All statistical analyses were performed using SPSS 26.0 (IBM Corp., Armonk). Missing data were minimal (<5%) across all variables and time points. Multiple imputation was performed using 5 imputed datasets, with the imputation model including all primary and secondary outcome variables (psychological capital, work engagement, and turnover intention) as well as baseline demographic covariates (age, sex, educational level, and work experience). Analyses were performed on each imputed dataset, and results were pooled using Rubin rules. All randomized participants were included in the ITT analysis, as detailed in the CONSORT 2010 flow diagram (Fig. 1). A two-tailed *P*-value <.05 was considered statistically significant.

## 3. Results

### 3.1. Baseline characteristics

Baseline demographic and professional characteristics were comparable between the intervention and control groups (all *P* > .05). No significant differences were observed in sex, age, educational level, marital status, work age, or professional title (Table 1), indicating adequate baseline equivalence.

### 3.2. Psychological capital (Table 3)

Significant group × time interaction effects were found for all dimensions of psychological capital and the total score (all *P* < .001).

**Table 3:** Comparison of Psychological Capital Scale scores in 2 groups at different time points.

	Group	T0 (pre-intervention)	T1 (post-intervention)	T2 (1 months post)	T3 (3 months post)	*F*	*P*	*η* ^*2*^	95% CI
Optimism	Intervention	8.79 ± 3.77	13.15 ± 3.12	12.83 ± 3.16	12.51 ± 3.22	42.86	<.001	0.26	0.16–0.36
	Control	8.82 ± 3.75	8.91 ± 3.70	8.87 ± 3.72	8.80 ± 3.76				
Self-efficacy	Intervention	25.85 ± 4.63	28.84 ± 3.93	28.52 ± 3.96	28.19 ± 4.03	18.35	<.001	0.13	0.06–0.22
	Control	25.88 ± 4.60	26.09 ± 4.56	26.03 ± 4.58	25.96 ± 4.61				
Resilience	Intervention	21.84 ± 3.43	24.95 ± 3.47	24.62 ± 3.50	24.29 ± 3.52	25.62	<.001	0.17	0.09–0.26
	Control	21.81 ± 3.45	22.03 ± 3.42	21.96 ± 3.44	21.89 ± 3.46				
Hope	Intervention	22.92 ± 4.34	29.11 ± 3.29	28.78 ± 3.32	28.45 ± 3.36	51.73	<.001	0.30	0.19–0.40
	Control	22.95 ± 4.32	23.10 ± 4.28	23.02 ± 4.30	22.95 ± 4.33				
Psychological Capital Scale score	Intervention	92.67 ± 12.89	107.39 ± 10.74	105.36 ± 10.83	103.24 ± 11.04	48.91	<.001	0.29	0.18–0.39
	Control	92.73 ± 12.85	94.02 ± 12.49	93.86 ± 12.53	92.91 ± 12.76				

*Note*: Pairwise comparisons: At T1 to T3, all scores in the intervention group were significantly higher than those in the control group (all *P* < .001); within the intervention group, T1 to T3 scores were significantly higher than T0 (all *P* < .001); within the control group, no significant differences at all time points (all *P* > .05).

Optimism: *F* = 42.86, *P* < .001, partial *η*^2^ = 0.26 (95% CI: 0.16–0.36).

Self-efficacy: *F* = 18.35, *P* < .001, partial *η*^2^ = 0.13 (95% CI: 0.06–0.22).

Resilience: *F* = 25.62, *P* < .001, partial *η*^2^ = 0.17 (95% CI: 0.09–0.26).

Hope: *F* = 51.73, *P* < .001, partial *η*^2^ = 0.30 (95% CI: 0.19–0.40).

Total psychological capital: *F* = 48.91, *P* < .001, partial *η*^2^ = 0.29 (95% CI: 0.18–0.39).

Compared with the control group, the intervention group showed significantly higher scores at T1, T2, and T3 (all *P* < .001). Within the intervention group, scores at T1 to T3 were significantly higher than at baseline (T0), while no significant changes were observed in the control group across time points.

### 3.3. Work engagement (Table 4)

Significant group × time interaction effects were detected for all dimensions of work engagement and the total score (all *P* < .001).

**Table 4:** Comparison of work engagement scores in 2 groups at different time points (mean ± SD, n = 61 per group).

	Group	T0	T1	T2	T3	F	*P*	*η* ^*2*^	95% CI
Vitality	Intervention	17.59 ± 3.21	25.19 ± 2.95	24.86 ± 2.99	24.52 ± 3.03	58.27	<.001	0.32	0.21–0.42
	Control	17.62 ± 3.19	17.87 ± 3.16	17.80 ± 3.18	17.73 ± 3.20				
Dedication	Intervention	14.03 ± 2.79	20.25 ± 2.83	19.92 ± 2.86	19.58 ± 2.89	49.83	<.001	0.29	0.18–0.39
	Control	14.05 ± 2.77	14.19 ± 2.74	14.12 ± 2.76	14.05 ± 2.78				
Focus	Intervention	16.67 ± 3.79	23.13 ± 3.04	22.79 ± 3.07	22.45 ± 3.10	55.19	<.001	0.31	0.20–0.41
	Control	16.69 ± 3.77	16.85 ± 3.74	16.78 ± 3.76	16.71 ± 3.78				
Work Engagement Scale score	Intervention	48.29 ± 8.98	68.57 ± 7.79	66.45 ± 7.96	64.29 ± 8.11	62.45	<.001	0.34	0.33–0.44
	Control	48.34 ± 8.95	49.10 ± 8.83	48.88 ± 8.78	48.70 ± 8.80				

SD = standard deviation.

Vitality: *F* = 58.27, *P* < .001, partial *η*^2^ = 0.32 (95% CI: 0.21–0.42).

Dedication: *F* = 49.83, *P* < .001, partial *η*^2^ = 0.29 (95% CI: 0.18–0.39).

Focus: *F* = 55.19, *P* < .001, partial *η*^2^ = 0.31 (95% CI: 0.20–0.41).

Total work engagement: *F* = 62.45, *P* < .001, partial *η*^2^ = 0.34 (95% CI: 0.23–0.44).

The intervention group exhibited significantly higher work engagement scores than the control group at all follow-up time points (all *P* < .001). Scores in the intervention group increased significantly from baseline to post-intervention and remained stable through the 3-month follow-up, whereas the control group showed no significant changes over time.

### 3.4. Turnover intention (Table 5)

A significant group × time interaction effect was observed for turnover intention (*F* = 89.62, *P* < .001, partial *η*^2^ = 0.43 [95% CI: 0.31–0.52]).The intervention group showed a marked and sustained reduction in turnover intention from baseline to T1, T2, and T3, with significantly lower scores than the control group at all post‑intervention time points (all *P* < .001). No significant changes were found in the control group across the study period.

**Table 5:** Comparison of turnover intention scores in 2 groups at different time points.

	Group	T0	T1	T2	T3	*F*	*P*	*η* ^*2*^	95% CI
Turnover Intention Scale score	Intervention	16.48 ± 3.06	10.61 ± 2.00	10.90 ± 2.11	11.22 ± 2.15	89.62	<.001	0.43	0.31–0.52
	Control	16.45 ± 3.08	16.31 ± 3.02	16.22 ± 3.00	16.17 ± 3.03				

### 3.5. Summary of intervention effects

All group × time interaction effects were statistically significant and showed moderate to large effect sizes (partial *η*^2^ ranging from 0.13 to 0.43). All 95% confidence intervals excluded zero, confirming stable, robust, and clinically meaningful intervention effects that persisted for at least 3 months.

## 4. Discussion

In the contemporary healthcare landscape, ED nurses constitute a distinct professional group, facing unique occupational challenges. These healthcare professionals bear critical responsibilities in managing acute and life-threatening conditions, operating within a high-pressure environment characterized by stringent standards, excessive workloads, and elevated occupational risks. The effective execution of their duties requires substantial psychological capital, which enables them to meet their professional obligations, commit voluntarily to patient care, and deliver precise and efficient medical interventions. The present study found that ED nurses exhibited psychological capital levels below the median range, a result consistent with meta-analytic evidence indicating that Asian nurses generally demonstrate lower levels of psychological capital compared to their counterparts in the United States and Australia. This regional disparity may be attributed to systemic factors, particularly nursing workforce shortages, which contribute to heightened occupational stress and negative workplace experiences.^[18,19]^ In their study, Hancock et al^[20]^ identified workplace violence and the complexity of nurse–patient relationships as unique sources of stress for nurses, distinguishing them from other medical staff. Research has shown that approximately 74% of nurses experience significant occupational stress.^[21]^ As a result, common psychological issues such as occupational burnout and turnover intention have emerged, severely impacting nurses’ physical and mental well-being, service quality, and patient safety.^[22–25]^ Thus, identifying and implementing effective strategies to enhance psychological capital among ED nurses has become a critical focus for healthcare administrators and researchers. The PCI-based approach, a widely adopted psychotherapeutic method in clinical settings, offers a modular thematic framework for structured interventions. This approach emphasizes fostering peer communication among nurses, allowing them to gain emotional comfort, social support, and professional recognition from their colleagues. Through the promotion of empathy and the use of diverse therapeutic techniques (including interactive games, psychodrama, case study analyses, role-reversal exercises, and in-depth interviews) the intervention facilitates profound self-awareness among nurses, while promoting deeper mutual understanding within teams. The findings of this study demonstrate that PCI-based intervention training significantly enhanced the psychological capital levels of ED nurses, a result consistent with the findings of Teng et al.^[26]^ Existing research has shown that well-established psychological interventions, such as mindfulness-based stress reduction and resilience training, can effectively reduce stress levels, mitigate burnout and emotional exhaustion, and enhance psychological capital.^[27–30]^ Therefore, implementing effective human resource management strategies and educational interventions is essential for mitigating nurse burnout and enhancing psychological capital among nursing professionals.

Research has also demonstrated that high levels of work engagement among nurses are significantly associated with improved professional identity, increased job satisfaction, enhanced work performance, better clinical nursing quality, and stronger patient safety outcomes.^[31]^ Conversely, low levels of work engagement among nurses can lead to declines in subjective well-being, professional identity, and work efficiency, ultimately compromising the quality of nursing care and potentially affecting patient safety and health outcomes.^[32]^ The findings of this study revealed relatively low levels of work engagement among ED nurses, a phenomenon likely attributable to the demanding workloads and high occupational stress they experience. These factors negatively affect their work motivation, leading to fatigue and impairing their ability to maintain focus, thereby diminishing engagement levels. The implementation of PCI-based intervention training led to a statistically significant improvement in work engagement among ED nurses (*P* < .05), aligning with findings reported by Pan et al.^[33]^ The intervention enabled nurses to gain comprehensive self-awareness regarding their strengths and weaknesses, while equipping them with strategies to overcome self-doubt. This enhanced their professional confidence, fostered an optimistic mindset, and facilitated adaptation to the work environment. Consequently, participants exhibited greater resilience in facing adversities, as well as increased work engagement, demonstrated by their heightened commitment to their professional responsibilities.

Enhancing nurse retention rates is a critical objective within the global healthcare sector. Empirical evidence from multiple studies indicates that a substantial proportion of surveyed nurses express intentions to leave their current positions.^[34]^ Studies have indicated a progressive increase in the prevalence of burnout syndrome among ED nurses,^[35,36]^ with mental health and emotional well-being emerging as significant contributing factors to nurse turnover.^[37–39]^ Peng et al^[40]^ found that nurses with high positive psychological capital naturally experience lower levels of burnout. The results of this study revealed that turnover intention scores among ED nurses were significantly lower after the intervention compared to baseline measurements (*P* < .05). This suggests that the PCI-based approach effectively reduced turnover intention, demonstrating superior efficacy in mitigating nurses’ propensity to leave their positions. The underlying mechanisms may be attributed to the PCI training’s emphasis on enhancing nurses’ sense of meaning and purpose in their professional roles. Specifically, the intervention fostered work-related mission awareness and self-efficacy, promoting positive self-identification that extended into nursing practice. These psychological improvements likely contributed to reduced occupational stress, increased job satisfaction, and ultimately decreased turnover intention.

The findings of this study indicate that psychological capital among ED nurses is significantly positively correlated with work engagement and significantly negatively correlated with turnover intention. These results align with findings reported by Xue et al^[41]^ and Tian et al,^[42]^ further highlighting the role of positive psychological capital as a key predictor of nurses’ work engagement. Specifically, nurses exhibiting higher levels of self-confidence, optimism, self-efficacy, and resilience are more likely to sustain work engagement. This can be explained by the fact that nurses with elevated psychological capital demonstrate greater persistence and resilience in the face of workplace challenges, rather than succumbing to frustration or resignation. These findings underscore the critical role of psychological capital in nursing practice. From a managerial perspective, these results suggest that nursing administrators should prioritize enhancing psychological capital among nursing staff as an effective strategy to improve work engagement. Such interventions could potentially contribute to workforce stability and enhance the overall quality of nursing care delivery.

## 5. Limitations

Although our study investigated the effects of PCI-based intervention training on 122 emergency nurses in a single tertiary hospital, several limitations should be acknowledged. First, the study was conducted at a single tertiary hospital in Zhejiang Province, and the sample was predominantly female, which may limit the generalizability of the findings to nurses in secondary hospitals, primary care institutions, or other regions of China. Future research should aim to include more diverse samples (e.g., nurses from different hospital tiers, male nurses) to enhance external validity. Second, the follow-up period was limited to 3 months; therefore, the long-term effects (e.g., 6–12 months) of the PCI intervention on actual nurse turnover behavior, rather than just turnover intention, need to be explored in extended follow-up studies. Third, this study did not examine the mediating or moderating mechanisms underlying the intervention’s effects (e.g., whether social support mediates the relationship between psychological capital and work engagement). Future studies could employ structural equation modeling to better understand these pathways. Fourth, while the sample size was increased to 122, it remains a single-center sample; therefore, multicenter studies with larger samples are recommended to further validate the intervention’s effects. Fifth, the study design may be susceptible to attention bias and the Hawthorne effect. The intervention group received structured, interactive, group-based psychological intervention with consistent peer interaction and emotional engagement, whereas the control group received only routine professional training. The enhanced interpersonal contact, structured support, and increased attention delivered in the intervention arm may have contributed independently to the observed improvements in psychological capital, work engagement, and turnover intention. Although an active psychosocial control condition was considered during the initial study design to control for nonspecific intervention effects, it was not implemented due to clinical feasibility constraints, ethical considerations regarding equitable resource allocation, and the intensive workload characteristics of emergency nursing practice. This potential limitation should be acknowledged when interpreting the causal specificity of the PCI intervention effects. Sixth, all outcome variables in this study, including psychological capital, work engagement, and turnover intention, were measured exclusively via self-report questionnaires, which may introduce common method bias. Although the validated scales used have demonstrated good reliability and validity, no objective indicators, administrative data, or observer-rated assessments were included to triangulate the findings. Furthermore, actual turnover behavior was not measured at the 3-month follow-up; only turnover intention was assessed. Future research should incorporate objective outcome data (e.g., actual turnover records, supervisor-rated performance, or clinical quality indicators) and use procedural or statistical remedies to reduce common method bias. Seventh, this study focused on longitudinal intervention effects using repeated-measures analysis rather than formal mediation testing. Future research with larger sample sizes should employ structural equation modeling or mediation analysis to clarify the potential mediating role of psychological capital in the relationship between PCI intervention, work engagement, and turnover intention. Eighth, although intervention fidelity was high (intraclass correlation coefficient = 0.92), fidelity assessment was based on review of 30% of recorded sessions rather than real-time observation of all sessions, which represents a minor limitation in reproducibility reporting.

## 6. Conclusion

This study demonstrates that a theory-based, 6-week psychological capital intervention can effectively improve psychological capital and work engagement, while reducing turnover intention among emergency nurses. The effects were consistent up to 3 months post-intervention, supporting the clinical applicability of PCI in emergency nursing settings.

Future research should focus on the following areas: extending the follow-up period to 1 year to evaluate the long-term effects of the intervention on actual turnover behavior; exploring the use of digital platforms (e.g., WeChat mini-programs, online courses) for delivering PCI interventions, which could improve accessibility for nurses with demanding work schedules; conducting multicenter studies with larger and more diverse samples to further validate the generalizability of the intervention’s effects; investigating the cost-effectiveness of the PCI intervention to provide comprehensive evidence to support its adoption by hospital administrators. This study contributes to the localized application of PsyCap theory in the field of emergency nursing in Chinese tertiary hospitals.

## Acknowledgments

The authors would like to appreciate the Medical and Health Science and Technology Project of Hangzhou, Zhejiang Province for supporting this research. We have got the permission (Table S1) for the use of the PCQ table in our manuscript from Fred Luthans.

## Author contributions

**Conceptualization:** Kun Sun, Xiaoyan Gong, Guoping Yuan.

**Formal analysis:** Kun Sun.

**Funding acquisition:** Kun Sun.

**Investigation:** Xiaoyan Gong.

**Project administration:** Guoping Yuan.

**Supervision:** Xiaoyan Gong.

**Validation:** Xiaoyan Gong.

**Writing – original draft:** Kun Sun.

**Writing – review & editing:** Kun Sun, Guoping Yuan.


